# Prevalence and Predictors of Depression among Pregnant Women in Debretabor Town, Northwest Ethiopia

**DOI:** 10.1371/journal.pone.0161108

**Published:** 2016-09-12

**Authors:** Telake Azale Bisetegn, Getnet Mihretie, Tefera Muche

**Affiliations:** 1 Department of Health Education and Behavioral Sciences, College of Medicine and Health Sciences, University of Gondar, Gondar, Ethiopia; 2 Department of Psychiatry, College of Medicine and Health Sciences, University of Gondar, Gondar, Ethiopia; 3 Amanuel Mental Specialized Hospital, Addis Ababa, Ethiopia; Xi'an Jiaotong University School of Medicine, CHINA

## Abstract

**Background:**

Depression during pregnancy is a major health problem because it is prevalent and chronic, and its impact on birth outcome and child health is serious. Several psychosocial and obstetric factors have been identified as predictors. Evidence on the prevalence and predictors of antenatal depression is very limited in Ethiopia. This study aims to determine prevalence and associated factors with antenatal depression.

**Methods:**

Community based cross-sectional study was conducted among 527 pregnant women recruited in a cluster sampling method. Data were collected by face-to-face interviews on socio-demographic, obstetric, and psychosocial characteristics. Depression symptoms were assessed using the Edinburgh Postnatal Depression Scale (EPDS). The List of Threatening Experiences questionnaire (LTE-Q) and the Oslo Social Support Scale (OSS-3) were used to assess stressful events and social support, respectively. Data were entered into Epi-info and analyzed using SPSS-20. Descriptive and logistic regression analyses were carried out.

**Results:**

The prevalence of antenatal depression was found to be 11.8%. Having debt (OR = 2.79, 95% CI = 1.33, 5.85), unplanned pregnancy (OR = 2.39, 95% CI = (1.20, 4.76), history of stillbirth (OR = 3.97, 95% CI = (1.67,9.41), history of abortion (OR = 2.57, 95% CI = 1.005, 6.61), being in the third trimester of pregnancy (OR = 1.70, 95% CI = 1.07,2.72), presence of a complication in the current pregnancy (OR = 3.29, 95% CI = 1.66,6.53), and previous history of depression (OR = 3.48, 95% CI = 1.71,7.06) were factors significantly associated with antenatal depression.

**Conclusion:**

The prevalence of antenatal depression was high, especially in the third trimester. Poverty, unmet reproductive health needs, and obstetric complications are the main determinants of antenatal depression. For early detection and appropriate intervention, screening for depression during the routine antenatal care should be promoted.

## Background

Depression during pregnancy is a prevalent mental health problem affecting about one in five women worldwide [1–4]. Antenatal depression is as prevalent in low and middle income countries if not more so as in high income countries [4]. It has been now recognized as a global public health problem owing to its severity, chronic nature and recurrence as well as its negative impact on the general health of women and development of children[5–9]. The prevalence of antenatal depression is estimated to be 15.6% in low and middle income countries although there is no evidence from 92% of the countries [4].

In Ethiopia, there is shortage of published report on the prevalence of depression during pregnancy. Few studies were published before the conduct of this study that reported prevalence estimates of antenatal depression [10] although more have been so recently [11,12]. The first study was conducted in the southern region of the country and used the Self Reporting Questionnaire (SRQ-20) with a cutoff point of 6 or more. Both of the other studies used the Edinburgh Postnatal Depression Scale (EPDS) with a cutoff point of 13 or more. The former was conducted in southwestern Ethiopia while the latter in the capital city, Addis Ababa.

### The risk and protective factors of antenatal depression in low and middle income countries

Several socioeconomic and psychosocial factors have been examined to be responsible for the development and maintenance of antennal depression. A systematic review by Fisher et al., described the determinants of perinatal depression into socioeconomic demographic, reproductive health, and psychosocial factors [4]. As expected taking the diversity of socio-cultural characteristics of the low and middle income countries, there are a lot of inconsistencies in the reports of risk and protective factors across and within countries [13]. The most consistently significantly associated factors were low income, rural residence, intimate partner violence, poor partner and social support, unintended pregnancy, and pregnancy complications [4]. Education, employment and strong partner and social support were found to be protective factors [4].

In Ethiopia, the most frequently cited determinants are similar to the factors reported in other low and middle income countries including rural residence, low education, low income and unmet needs in reproductive health and obstetric care. The available studies indicate that unplanned pregnancy, lack of partner support[11,12], and household food insecurity[12]were found to be strong predictors of antenatal depression. The impact of untreated antenatal depression on pregnancy outcome and child health and development has been studied in many low and middle income countries [14–16]. Two studies examined the impact of antenatal depression on birth outcome. One of them found an association between antenatal depression and low birth weight[17] while the other didn`t[18]. In the second study, prolonged labor and delayed initiation of breast feeding were associated with antenatal depression [18]. The prevalence, the risk factors and consequences of antenatal depression are not widely studied in low and middle income countries. Moreover, the available studies have inconsistent findings including the Ethiopian studies. Ethiopia is a low income country with very diverse ethnic and socio-cultural characteristics that could add to the inconsistency of reports in prevalence and risk factors of antenatal depression. Therefore, this study was aimed to determine the prevalence and determinants of antenatal depression in one of the zonal towns of the northwestern region.

## Methods

This community based cross-sectional study was conducted at Debretabor town between April and May 2013.The study area, Debretabor town is the capital of South Gondar zone, one of the 11 zones of the Amhara regional state. It is 97 kilometers to the east of Bahrdar the capital of the state. It is about 667 km north of the capital city Addis Ababa. According to South Gondar zone catchment profile, the total population of the town was estimated to be 67543 of which 49.8% are females as of 2013. The town is sub divided by eight *Kebeles* (lowest administrative unit). There were 11 health facilities (1 hospital, 3 health centers, 4 health posts & 3 private clinics) providing health services during the data collection period.

Antenatal care is one of the services rendered within the town. ANC activities are carried out by health extension workers who are assigned in each Kebele (sub-district). Within each Kebele there are 3–4 health extension workers. According to the district health office report of the previous year, the proportion of pregnant women who used antenatal service for four or more times was 65.5%[19].

### Sampling

All pregnant women listed by the health extension workers were included in the sampling frame. A cluster sampling was used to select 543pregnant women from four sub-districts (kebeles) out of the total eight.

Participants were identified by obtaining official lists of the pregnant women from health extension workers working in the area, who routinely collect data on new pregnancies.

### Measurements

Antenatal depression was assessed using Edinburgh Postnatal Depression Scale (EPDS). The EPDS was validated in Ethiopia among postnatal women and gave a sensitivity and specificity of 78.9% and 75.3% respectively[20]. A score of 12 or more in the Edinburg Postnatal Depression Scale was considered depression in this study. However, the EPDS was not a good measure of CMD for rural setting. We used a higher cut-off point as the study area is not comparable to Addis Ababa, although it is an urban setting.

Experience of stressful life events during the six months period prior to assessment was assessed using the List of Threatening Experiences (LTE)[21]. The 12 items were categorized into five categories namely health risks, loss of a loved one, relationship difficulties, income instability, and legal problems[22]. The LTE contains 12 categories of significant life events, for example relating to death of close persons, loss of relationships, imprisonment, and loss of valued object. These 12 categories accounted for two thirds of all events collected in the original development of the tool. The LTE has good test-retest reliability (Kappa: 0.61–0.87) and predictive validity[23]. Social support was measured by the Oslo 3-item Social Support Scale (OSS)[24]. The social support scores were categorized into poor or no social support for scores less than nine. The scores from 9–14 were considered moderate to strong support and merged together as “yes” for social support.

The OSSS-3 contains three items assessing the number of close confidants, perceived level of concern from others and perceived ease of getting help from neighbors. The OSSS has good convergent and predictive validity[25]. Both the list of threatening experiences (LTE-12) and the Oslo Social Support Scale (OSSS-3) have been used in a population level study in Ethiopia [26]

### Data collection

Data were collected using the Amharic version of a semi structured questionnaire which included socio-demographic, obstetric and psychosocial variables. Four nurses with diploma working at Debretabor hospital were recruited and trained for two days as data collectors. They were available at each site on a daily basis to interview the participants in their homes.

Two nurses with bachelor degree were used as supervisors and were roving in each site to assist and supervise the data collectors.

Pretest was done at a nearby district on 30 pregnant women to check clarity of the instrument. Based on the finding from the pretest, the questionnaire was revised and adapted. The English version of the questionnaire was translated into Amharic and then back into English to maintain its consistency. The collected data were checked daily for completeness and consistency.

### Data analysis

Data were entered, cleaned and analyzed using SPSS version 16. Descriptive statistics (frequencies, percentage, means and standard deviations) were performed.

Bivariate analysis was conducted to assess the relationship between each independent variable and the outcome variable (antenatal depression). To control for the effect of confounding factors, multivariate logistic regression was constructed including variables with p-value of 0.2 or less in the bivariate analysis. The degree of association between dependent and independent variables were assessed using odds ratio with 95% confidence interval.

### Ethical issue

Ethical approval was obtained from University of Gondar and Amanuel Mental Specialized Hospital ethical review board. Permission was received from Debretabor town health office. A written informed consent was obtained from each participant following provision of an outline of the purpose of the study. For participants who were unable to read and write, a family member or data collector read the information sheet. If they agreed to participate in the study, they would give a finger print. Confidentiality was maintained by using anonymous questionnaire and privacy was assured.

## Results

Of the 543 women invited to participate in the study, 527 agreed, yielding a response rate of 97%.The age of participants ranged from 17 to 39 years (mean = 27.5(SD, ± 4.95).

More than a quarter 143(27.1%) were unable to read and write; 458(86.9%) were married; 88(16.7%) had experienced hunger in the month prior to interview 89(16.9%) had debt to buy food (**Table 1**).

**Table 1:** Frequency distribution of Socio-demographic and socio-economic factors among pregnant women at Debretabor town, North West Ethiopia, 2013(n = 527).

Characteristics	Frequency	Prevalence rate (%)
**Age group**		
≤19	24	4.6
20–24	121	23.0
25–29	202	38.3
30–34	115	21.8
≥35	65	12.3
**Education**		
No formal education	198	37.5
Primary	112	21.4
secondary	114	21.6
More than secondary	103	9.5
**Occupation**		
Housewife	290	55
Government Employee	121	23
Student	116	22.0
**Religion**		
Orthodox	406	77.0
Muslim	96	18.2
Protestant	25	4.7
**Ethnicity**		
Amhara	511	97.0
Oromo	10	1.9
Tigre	6	1. 1
**Marital status**		
Not currently married	69	13.1
Married	458	86.9
**Hunger in the past month**		
Yes	88	16.7
No	439	83.3
**Debt**		
Yes	89	16.9
No	438	83.1

One hundred eleven (21%) reported one or more pregnancy related diseases (hypertension, anemia, diabetes mellitus, or bleeding) had a complication during the current pregnancy. History of still birth was reported by 66 (12.5%) of the participants. The current pregnancy was unplanned for 160(30.4%) of the participating women.

One or more stressful event in the preceding six months was reported by 156(29.6%) of the respondents. More than one-third, 198(37.6%) of the participants reported that the social support they receive was poor.

### Prevalence of antenatal depression

Overall, participants who scored above the cutoff point in the EPDS were 62(11.8%) indicating possible depression. Depressive symptoms were reported by 8/87(9.2%) 13/175(7.4%) and 41/265(15.5%) of pregnant women in their first, second and third trimester of pregnancy respectively.

Demographic variables such as age, education and occupation were not associated with antenatal depression. In the multivariable logistic regression analysis, the odds of having antenatal depression were higher for those who owed money. Women whose current pregnancy was unintended were more than twice more likely to have depression than those who planned their pregnancy. Previous adverse pregnancy outcomes of stillbirth and abortion increased the odds of antenatal pregnancy. Being in the third trimester of pregnancy had increased odds of depression. The odds of depression were almost three times higher in those who gave history of depression. **(Table 2).** Among the five categories of stressful experiences health risk which includes illness, injury, or assault to the subject or a close relative, was strong predictor of antenatal depression. **(Table 3)**

**Table 2:** Demographic and obstetric factors associated with depression among pregnant women at Debretabor town, North West Ethiopia, 2013.

Characteristics	Antenatal Depression		Crude Odds Ratio (95%CI)	Adjusted Odds Ratio) 95%CI)
	Yes	No		
**Age**				
20–24	13	109	0.34(0.148, 0.76)	0.56(0.17, 1.82)
25–29	22	180	0.37(0.18,0 .76)	0.64 (0.22, 1.818)
30–34	11	104	0.32(0.14,0 .75)	0.45(0.14, 1.40)
≥35	16	49	1	1
**Education**				
No formal education	32	166	1.71 (0.37, 7.91)	1.02 (0.95, 1.09)
Primary	10	102	1.44 (0.27, 7.50)	0.46 (0.06, 3.45)
Secondary	9	105	0.83 (0.16, 4.13)	0.41 (0.04, 3.46)
More than secondary	11	92	1	1
**Hunger in the past one month**				
Yes	29	59	6.05(3.42,10.67)	1.70(.62,4.64)
No	33	406	1	1
**Debt**				
Yes	28	61	5.45(5.45, 9.62)	2.79(1.33,5.85)**
No	34	404	1	**1**
**Unplanned pregnancy**				
Yes	28	339	0.31(0.17,0.52)	**2.39 (1.20, 4.76)** *
No	34	126	1	**1**
**History of Still birth**				
Yes	22	44	5.57(2.84,10.90)	3.97(1.67,9.41)**
No	22	245	1	**1**
**History of Abortion**				
Yes	20	45	4.52(2.30, 8.85)	2.57(1.005, 6.61)*
No	24	244	1	**1**
**Trimester**				
1^st^ trimester	8	79	1	1
2^nd^ trimester	13	162	0.79(0.31, 1.99)	1.27 (.40, 3.97)
3^rd^ trimester	41	224	1.81(.81, 4.02)	1.70(1.07,2.72)*
Complication during current pregnancy				
Yes	30	81	4.44(2.55, 7.72)	3.29(1.66,6.53)**
No	32	484	1	**1**
**Fear of pregnancy**				
Yes	37	15	2.93(1.70, 5.04)	1.32 (.63, 2.73)
No	25	309	1	1
**Previous history of depression**				
Yes	33	91	4.68(2.7 0,8.09)	3.48(1.71,7.06)**
No	29	374	1	**1**
**Family history of depression**				
Yes	14	60	1.97(1.02,3.78)	0.89(0.35,2.20)
No	48	405	1	1

* significant at p-value < 0.05

** significant at p-value <0.01

**Table 3:** Bivariate and multivariate analysis of psychosocial factors (stressful events and social support) and their association with depression among pregnant women at Debretabor town, North West Ethiopia, 2013.

Characteristics	Antenatal Depression		Crude Odds Ratio at 95%CI	Adjusted Odds Ratio at 95%CI
	Yes	No		
**Categories of stressors (LTE)Health risk**				
Yes	31	111	**3.19(1.856,5.481)**	2.04 (1.01,4.11)*
No	31	354	1	**1**
**Death loved one**				
Yes	25	141	1.55(0.90,2.67)	1.09(0.53,2.25)
No	37	324	1	1
**Income instability**				
Yes	42	158	4.08(2.31,7.18)	1.91(0.94,3.84)
No	20	307	1	1
**Relational problem**				
Yes	26	101	2.60(1.50,4.51)	1.52 (0.74,3.09)
No	36	364	1	1
**Legal issues**				
Yes	32	122	2.99(1.74,5.14)	1.94(0.96, 3.91)
No	30	343	1	1
**Social support (OSSS-3)**				
Yes	39	159	3.26(1.88,5.65)	1.57 (0.79,3.11)
No	23	306	1	1

*significant at p-value < 0.05

LTE: List of Threatening Experiences, OSSS-3: Oslo Social Support Scale, 3 items

## Discussion

In the present study the prevalence of antenatal depression was 11.8%, and prevalence rates at first, second, and third trimesters were 9.2%, 7.4%, and 15.5% respectively. This finding was similar with a systematic review conducted in high income countries which reported 7.4%, 12.8%, and 12%[27]. Compared to estimates of other low and middle income countries, this finding is lower [4]. It is also lower than the prevalence reports from other regions of Ethiopia such as Addis Ababa[11] and southwestern Ethiopia[12] but similar to a study conducted in Butajira, south Ethiopia[10]. In small towns in Ethiopia such as this study area where people from rural parts of the country settle, traditional practices including the supports provided to perinatal women seem to be maintained. These socio-cultural practices have protective effect against depression [28]. None of the socio-demographic variables was found to be significantly associated with antenatal depression. This is contrary to other studies where younger age, rural residence, low education, and unemployment were strong predictors of antenatal depression [4]. The inability of these variables to predict antenatal depression may be explained by the homogeneity of the population in terms of life styles between the urban and rural, education and employment where most of the respondents of this study are below high school level. Pregnant women with debt were 2.79 times more likely to experience antenatal depression as compared to those pregnant women who had no debt. The result was consistent with the findings from Pakistan [29], South Africa [30,31], and Brazil [32]

In this study, pregnant women who had not planned their current pregnancy were 2.39 times more likely to have antenatal depression than those who planned their pregnancy. This finding is in line with studies in other LMICs [4,33] and studies in other parts of Ethiopia [11,12].

Pregnant women who have history of still birth were 3.97 times more likely to have antenatal depression than pregnant women who have no history of still birth. This was supported by studies conducted in other LMICs [4,34]. In this study, antenatal depression was 2.57 times more in women who have history of abortion than who had not history of abortion. This finding was consistent with a study in Brazil [35]. Those women in their third trimester of pregnancy were 1.70 times more likely to experience antenatal depression than women in their first trimester of pregnancy. This was in line with a systematic review conducted in high income countries [27]

Current pregnancy complication was significantly associated with antenatal depression. The result was similar with studies done in other LMICs [4]

In line with studies from other LMICs [4] and a study in Addis Ababa [11], past history of depression was significantly associated with antenatal depression. Those women who had a previous history of depression were about 3 times more likely to have antenatal depression as compared to those who had no history of past depression.

In this study, the experience of stressful event related to injury and illness during the six months prior to the interview was significantly associated with antenatal depression. The result was supported by other studies[36].

### Limitations of the Study

This is a cross-sectional study which tried to see the various factors that may predict antenatal depression. However, temporal relationship cannot be inferred using such a design such as poverty and depression. Measuring income in a population where most of the residents do not have specific wages is very difficult. Assessment of past history of depression and family history of depression were made by a case vignette. However, the concept of depression and the symptoms may be difficult to understand for the women. Moreover, there may be a recall bias. Although the Edinburgh Postnatal Depression Scale was validated in Addis Ababa, it was not found to be valid in rural areas. The study area for this research is a zonal town compared to Addis Ababa which is the capital city of Ethiopia

## Conclusion

Antenatal depression is a major public and mental health problem owing to its high prevalence. Poverty, unmet needs in reproductive health, and past history of mental illness were strong predictors of antenatal depression. Improving reproductive health and obstetric services as well as routine screening of women during pregnancy may be helpful to reduce the burden of antenatal depression.
